# What do measures of gender identity tell us about gender identity over time?

**DOI:** 10.1111/bjdp.12491

**Published:** 2024-05-07

**Authors:** Ellena Fisher, Sarah Wright, Cora Sargeant

**Affiliations:** ^1^ School of Psychology University of Southampton Southampton UK

**Keywords:** gender development, gender diversity, gender identity, longitudinal, measures, narrative synthesis, systematic review, transgender

## Abstract

Gender identity is a multifaceted concept and is represented by a wide range of measures and constructs including both self‐report and researcher observations of preferences and behaviours. However, despite their similar theoretical underpinning, gender identity measures are rarely found to correlate with one another, and contrasting patterns and trajectories are often found for each construct (Egan & Perry, *Developmental Psychology*, **37**, 2001, 451). Therefore, this systematic review aimed to present a review of the longitudinal research evidence surrounding gender identity development in the absence of formal intervention. Using a systematic search strategy, 21 studies were identified. Narrative synthesis was used to synthesize the data collected in these studies and trajectories were explored for (1) self‐identification measures of gender identity, (2) clothing preferences, (3) peer preferences, and (4) object/activity preferences. Overall, the results of this systematic review are consistent with wider research suggesting that distinct developmental patterns can be observed when using different constructs and measures of gender identity.

**Key points**

- The results of this systematic review are consistent with wider research suggesting that distinct developmental patterns can be observed when using different constructs and measures of gender identity.

## INTRODUCTION

### What is gender identity?

Gender‐diverse individuals have been present throughout history and experiences outside of the gender binary are found in cultures around the world (Devor, 1997; Herdt, 1994; Tompkins et al., 2015). However, contrasting assumptions about the nature of ‘gender’ have led to the pathologisation of gendered expressions that do not align with Westernized cultural stereotypes (Tompkins et al., 2015). Over time, there has been a move towards an affirmative model of care for gender‐diverse individuals (American Psychological Association (APA), 2015) and practitioners have been encouraged to recognize and accept varied gender expressions as a part of human diversity (Horton, 2020). However, conflicting theories surrounding gender identity development in children have led to ongoing worldwide debate about how best to support transgender children and young people (McClure et al., 2022).

There are a range of underlying assumptions about what ‘gender’ means and this can vary based on cultural and societal norms. Morgenroth and Ryan (2018) argue that conceptual understandings of gender fall into 3 categories: (1) evolutionary understandings based on biological sex, (2) social structural approaches based on societal norms and structures, and (3) social identity approaches based on gender as a social category with which an individual identifies. Across the research literature, ‘sex’ is often used to refer to the biological characteristics of an individual (e.g., chromosomes), while ‘gender’ typically refers to the societal and cultural meanings associated with femininity and masculinity (Lindqvist et al., 2021). The term ‘transgender’ is operationalized by Lindqvist et al. (2021) as ‘individuals whose assigned gender at birth does not correspond to their self‐defined gender identity’. These ‘assigned genders’, given to infants at birth, are often based on a dichotomous binary categorization of gender and sex. Some cultures hold on to the belief that gender is a binary system and there are distinct differences between men and women. This can lead to discrimination, self‐stereotyping, stereotype threat and institutional biases (Hyde et al., 2019).

With rising numbers of individuals whose experience cannot be defined using these binary categories, theories of gender identity have broadened, reflecting ideas of gender as a spectrum (Gülgöz et al., 2022) with multiple co‐occurring constructs (Ho & Mussap, 2019). These perspectives date back to earlier works by Butler (1990) and West and Zimmerman (1987), who argued that ‘gender’ is a performance that we ‘do’ repeatedly based on societal stereotypes, reinforcing an illusion of binary sex. Viewing gender as a societal construct rather than a characteristic determined by biological sex has led to an affirmative model of care being used internationally with gender‐diverse individuals, supporting an individual to consolidate their expressed gender (APA, 2015). These models are based on research suggesting that societal expectations and pressure to conform to gender norms have a negative impact on psychological outcomes (Egan & Perry, 2001; Hidalgo et al., 2013). Recent research supports this idea, with transgender children who have been affirmed in early childhood demonstrating improved levels of well‐being (Austin et al., 2022; Olson et al., 2016; Russell et al., 2018). Consequently, there has been significant criticism of models that attempt to influence children to accept their gender assigned at birth.

### Measures of gender identity

In line with broadening theories and models of gender, a diverse range of measures have been developed to attempt to understand the multifaceted nature of gender identity. Bates et al. (2022) note that these measures often focus on a person's internal understanding of their own gender, as well as their gender expression and how this is perceived by others. Wood and Eagly (2015) describe a biosocial model of gender in which an individual's gendered psychological attributes ‘emerge flexibly from a dynamic interaction among biological and social factors’. Reflecting this complexity, researchers frequently use a combination of measures, based on self‐report and observational methods. This systematic review of the literature aims to explore trajectories and patterns of gender identity development that are collected when using this range of measures and to investigate whether these measures correlate with one another.

#### Self‐identification

Self‐identification measures of gender identity are based on the descriptive categorization of self rather than interpretative accounts of personality or behavioural traits (Wood & Eagly, 2015). An individual is thought to develop a summary judgement of their overall gender identity based on attributes and preferences and the salience each of these attributes holds (Spence, 1993). To self‐identify as part of a group, an individual must be able to reflect on the features of this collective identity and categorize themselves as a group member (Turner et al., 1987). Contributing to this overall summary judgement are concepts such as gender typicality and gender contentment (Ashmore et al., 2004; Egan & Perry, 2001). Rather than relying on the assumptions and judgements of researchers, participants engage in self‐stereotyping, identifying gendered in‐group attributes in oneself as well as differences from the outgroup (Turner et al., 1987) to label themselves and report directly on their feelings of group membership (Wood & Eagly, 2015). For this reason, many researchers have attempted to explore the age at which a child is able to recognize their own gender and notice the gender of others in order to make this ‘summary judgement’, suggesting that children generally are able to label their own gender by the age of 2 (Thompson, 1975). When asked to label the gender of others, children have been found to justify their choices using physical gendered cues such as hair or clothing choices (Conn & Kanner, 1947).

An important consideration, in relation to self‐defined gender, is that a binary, dichotomous system does not reflect the experiences of gender‐fluid and non‐binary individuals. Over time, developments in psychological understanding regarding gender identity have led to a movement away from bipolar measures in which an individual is measured on a scale from masculine to feminine (Bem, 1974; Spence et al., 1975). Martin et al. (2017) proposed a dual identity model of gender identity, finding empirical support for the use of both same‐gender felt similarity and other‐gender felt similarity. Children were found to be able to use their own and other gender similarities across three time points, lending support to the use of 2 separate measures for a child to assess how much they ‘feel like a boy’ and also how much they ‘feel like a girl’, rather than placing these constructs on a single bipolar scale.

#### Intergroup bias

Another means of understanding a child's gendered experience is to explore sex segregation or intergroup bias. This is referred to by Kornienko et al. (2016) as a ‘between‐gender’ measure. Social identity theory (Tajfel et al., 1979) suggests that members of any group see their group as distinct from outgroups and the sense of belonging felt by a group member will automatically lead to in‐group bias. For this reason, researchers often use measures of group‐related judgements (e.g., ingroup bias or out‐group derogation) as a measure of gender identity (Wood & Eagly, 2015).

#### Gender stereotyped preferences

With some children not developmentally able to self‐identify at an early age, many researchers also use observational methods or parental reports of children's stereotyped preferences (Halim et al., 2013; Hässler et al., 2022; Lauer et al., 2018). Modern models of gender identity are based on early research suggesting that separate dimensions of masculinity and femininity are useful tools in understanding overall gender identity (Broverman et al., 1972). The personality traits and preferences associated with each of these dimensions are universally accessible and used frequently by social perceivers. Much of this traditional research into stereotypes focuses on agentic and communal traits because of their cultural associations with femininity and masculinity (Wood & Eagly, 2015).

In practice, gender‐stereotyped preferences are often measured using researcher observation of toy/activity preferences, as well as the clothing that a child chooses to present themselves to others. Gender expression is interwoven with social gender, focused on stereotyped and culturally specific demonstrations of femininity and masculinity (Lindqvist et al., 2021). For young children, these observable preferences and their congruence with gendered stereotypes can be a particularly salient way of self‐judging their gender‐typicality (Egan & Perry, 2001).

Despite the widespread use of the use of gender‐stereotyped preferences as a measure of gender identity, there are potential limitations of this type of measure. First, these preferences are typically based on a binary view of gender and do not acknowledge that some individuals experience more fluid or nonbinary gender identities (Diamond, 2020). The assumption is also made that an individual's gender expression is consistent with their gender identity (Geist et al., 2017). Non‐conformity to societal stereotypes in terms of gendered preferences is not always predictive of a transgender identity. Instead, adaptive and flexible use of gendered behaviours has been found to be associated with positive psychological adjustment (DiDonato et al., 2012).

#### Correlation between measures

Extensive literature has emerged that seeks to understand the trajectories of these interwoven aspects of gender development, investigating the stages at which a child is able to self‐identify and the patterns of their gendered preferences. However, gender identity measures are rarely found to correlate with one another, and distinct patterns and trajectories are often found for each construct (Bruun & Farr, 2020; Egan & Perry, 2001; Gülgöz et al., 2022; Halim et al., 2013; Hässler et al., 2022). Furthermore, despite many of these measures being developed around a binary system, many people do not demonstrate consistency across measures of masculinity and femininity, particularly when self‐reporting (Egan & Perry, 2001). Joel et al. (2015) investigated the internal consistency of gendered personality traits, attitudes, and behaviours. They found that it was very rare for an individual to demonstrate traits consistent with one gender and, instead, most humans possessed both masculine and feminine psychological characteristics (Joel et al., 2015; Spence, 1993). Hyde (2005) proposed the gender similarities hypothesis which states that men and women are very similar on most, but not all, psychological variables. Therefore, it may be dangerous to assume an overall gender identity from a single domain (Egan & Perry, 2001). A greater awareness of how an individual's gender identity develops over time, as evidenced by a combination of measures, may support a more nuanced understanding of gender identity development.

## METHOD

### Research questions

This systematic review was conducted with the aim of exploring the trajectories of gender identity development over time in cisgender and gender‐diverse individuals. The following research questions were explored:

*R1*: What do distinct categories of measures of gender identity tell us about trajectories of gender identity development?

*R2*: How do these trajectories fit together and what does this tell us about the overall development of an individual's gender identity?

### Search strategy

Initial scoping searches were conducted in January 2022. Five electronic databases were chosen: PsycINFO, ERIC, Web of Science (Core Collection), ProQuest Dissertations (Theses), and Scopus. Final searches were undertaken in June 2022 and search results were important in Rayyan systematic review software (see Table 1 for full details of the search strategy).

**TABLE 1:** Search strategy and associated results table.

Database	Search strategy	Number of results
Psych Info	**S1:** (Transgender* OR “gender identity” OR “gender identity disorder” OR “gender dysphoria” OR “gender divers*” OR “gender non‐conform*” OR transsexual OR transexual OR “gender typed preferences”) **S2:** (Longitudinal OR “long term” OR “lagged*” OR “follow up” OR “follow‐up” OR cohort OR “over time”) **S3:** S1 AND S2	3092
ERIC	**S1:** (Transgender* OR “gender identity” OR “gender identity disorder” OR “gender dysphoria” OR “gender divers*” OR “gender non‐conform*” OR transsexual OR transexual OR “gender typed preferences”) **S2:** (Longitudinal OR “long term” OR “lagged*” OR “follow up” OR “follow‐up” OR cohort OR “over time”) **S3:** S1 AND S2	111
Web of Science (Core collection)	Searching within abstracts and filtered to be articles. **S1:** (Transgender* OR “gender identity” OR “gender identity disorder” OR “gender dysphoria” OR “gender divers*” OR “gender non‐conform*” OR transsexual OR transexual OR “gender typed preferences”) **S2:** (Longitudinal OR “long term” OR “lagged*” OR “follow up” OR “follow‐up” OR cohort OR “over time”) **S3:** S1 AND S2	2059
ProQuest Dissertations and Theses	Anywhere except full text (NOFT), doctoral theses only **S1:** (Transgender* OR “gender identity” OR “gender identity disorder” OR “gender dysphoria” OR “gender divers*” OR “gender non‐conform*” OR transsexual OR transexual OR “gender typed preferences”) **S2:** (Longitudinal OR “long term” OR “lagged*” OR “follow up” OR “follow‐up” OR cohort OR “over time”) **S3:** S1 AND S2	413
Scopus	Search within abstract/title/keywords, filtered to be articles. **S1:** (Transgender* OR {gender identity} OR {gender identity disorder} OR {gender dysphoria} OR {gender divers*} OR {gender non‐conform*} OR transsexual OR transexual OR {gender typed preferences}) **S2:** (Longitudinal OR {long term} OR “lagged*” OR {follow up} OR {follow‐up} OR cohort OR {over time}) S3: S1 AND S2	4304

### Exclusion/inclusion criteria

Exclusion and inclusion criteria were used to assess the appropriateness and relevance of studies (see Table 2). Included studies were required to use a longitudinal or follow‐up study design with at least two clear time points and the use of a measure of gender identity. Studies were excluded where participants had been the recipient of some form of intervention to ensure that the included studies included measures of participants' naturally occurring gender development over time, without the influence of hormonal or psychological intervention. Social transition (e.g., changes in gender expression, name and/or pronoun) was not considered a formal intervention.

**TABLE 2:** Inclusion and exclusion criteria.

Study item	Inclusion criteria	Exclusion criteria
Type of research	Peer‐reviewed published papers and theses	Book chapters, systematic reviews, dissertations
Methodology	Qualitative, quantitative, or mixed‐methods longitudinal studies	Retrospective studies, or studies without at least 2 time points
Participants	Participants of all age ranges	
Intervention	Studies where participants were not subject to any formal intervention (e.g., hormonal, or psychological) The social transition was not considered a formal intervention.	Participants were subject to a formal intervention
Outcomes/Focus	A quantitative or qualitative measure of gender identity is used	No measure of participant gender identity
Language	Paper accessible in English	Paper not accessible in English
Date	All dates of publication	

### Screening and selection

**FIGURE 1:**
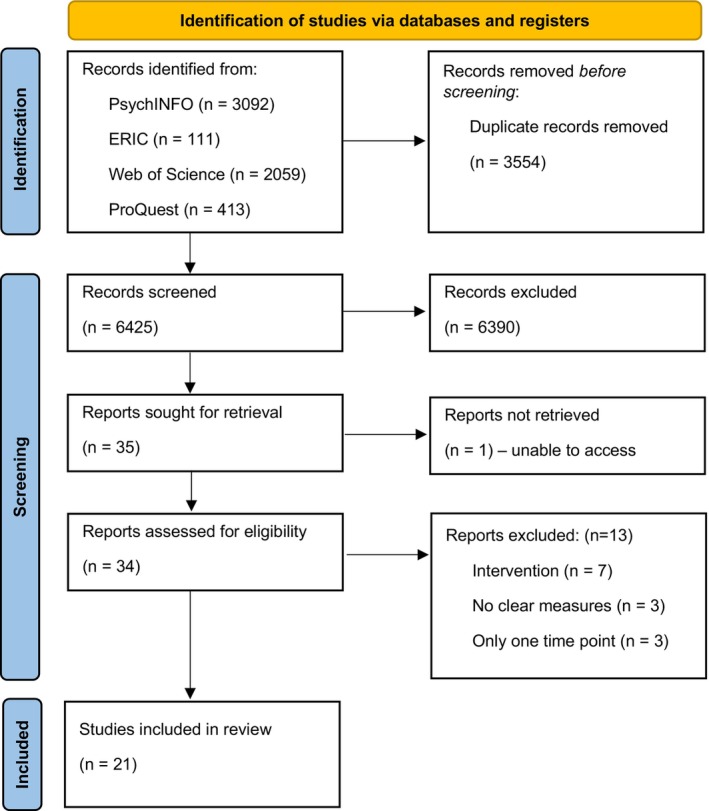
PRISMA 2009 flow diagram (Moher et al., 2009) of the systematic search process.

From the five databases, 9979 results were collected, and 3554 duplicates were removed. Title searching was then completed to assess the suitability of the remaining papers and 6378 were removed. A further 12 studies were excluded using abstract searching, leaving 35 papers selected for full‐text review. These 35 articles were double‐screened by members of the research team to ensure consistency and any disagreements were resolved through discussion. Of these 35 studies, 21 studies were selected for inclusion (see Figure 1 for PRISMA flow chart depicting this process).

### Quality assessment

Study quality was assessed using the Quality Assurance of Diverse Studies (QuADS) checklist, a revision of the QATSDD (Harrison et al., 2021). Specific criteria are provided alongside the QuADS to support the rating given to each study for each component and studies were given a percentage score that reflected the overall quality of the study. However, overall percentages were not used to determine inclusion or exclusion as there is no evidence to suggest that any of the criteria included in the QuADS checklist are more important than another and therefore it is not possible to decipher a ‘cut off point’ for inclusion (Harrison et al., 2021). The focus of the quality assessment, therefore, was to consider components of the included studies in relation to the specific research questions.

### Data extraction and analysis

Key characteristics and findings from each of the included studies were extracted and summarized in Appendix 1. Once data had been extracted, studies were grouped according to the following categories of measures: (1) self‐identification, (2) object and activity preferences, (3) clothing preferences, and (4) peer preferences. Results were collated for each study under these categories and then combined to make comments on the overall trajectory evidenced by studies using each category of measure. For each category of measure, results were extracted from studies that had collected data over time specific to this category. Studies in which it was not possible to isolate data specific to the category of measure were not included in the results. For example, it was not possible to include specific results under each category for studies in which researchers combined data collected across multiple categories of measures to make a judgement about a child's overall gender conformity (e.g., Barron & Capous‐Desyllas, 2017; DiDonato et al., 2012; Martin, 2010; Warin, 2000). Following analysis of individual measures of gender identity, these trajectories were compared to one another and integrated to make comments on gender identity development as a whole.

## RESULTS

### Study characteristics

All studies followed the gender identity of participants over time and used either a longitudinal or follow‐up study design. The time between initial data collection and the second time point ranged from 1 to 13 years. The most recent study was published in 2022 and the oldest study in 1989. All included studies were conducted in either the USA (*n* = 15), UK (*n* = 5), The Netherlands (*N* = 1) or Canada (*n* = 2). The majority of studies used quantitative methodology (*n* = 18), with 2 entirely qualitative studies and 1 mixed methods study. Sample sizes ranged from 4 participant families to 20,745 individual participants and included participants aged between 3 months and 32 years old. The focus of the studies was spread across early childhood (*n* = 8), middle childhood (*n* = 8), adolescence (*n* = 4), and adulthood (*n* = 1).

### Self‐identification

Self‐identification measures were used in many of the included studies, with participants across age groups. Four studies focused on self‐identification as their primary gender identity measure (Campbell et al., 2000; Gülgöz et al., 2022; Hässler et al., 2022; Shirley, 2000). Campbell et al. (2000) used the rouge mirror test, as outlined by Amsterdam (1972), to assess self‐identification in infants at 6, 9, and 18 months. At all stages of this study, the infants did not show signs of self‐recognition. This rouge test was also used by Shirley (2000) alongside an additional task where children were shown paired photographs of themselves and a same‐sex, same‐age peer to assess preferential looking. At 18 months, infants were found by Shirley (2000) to demonstrate self‐recognition but their performance on another gender labelling task indicated that their formal recognition of gender was not yet developed at 18 months.

Many of the included studies, in which a child is asked to self‐identify in this way, focused on middle childhood. Hässler et al. (2022) used self‐identification measures of gender similarity and gender identity to allow 7‐ and 9‐year‐olds to express their gender identity. Across the 2.5‐year time span, transgender and cisgender children's self‐identified gender remained stable across both measures. Gülgöz et al. (2022) used a gender spectrum continuum, ranging from ‘feel totally like a boy’ to ‘feel totally like a girl’ as a means of allowing children to express their gender identity. In the longitudinal part of this study, results showed moderate stability in the way that participants self‐identified using this spectrum measure over the 1–2‐year time period.

### Object and activity preferences

Fourteen of the studies investigated object and activity preferences as part of their measures of gender identity, beginning in very early childhood by observing the gaze of babies as young as 3 months old. Campbell et al. (2000), used pairs of gender‐congruent and‐gender incongruent stereotyped toys and activities to assess infant preferences over time. Overall, gendered preferences remained constant from 3 to 18 months (Campbell et al., 2000). Lauer et al. (2018) also found this predictive association between object preferences at 6 months of age and 4 years of age. Gender‐stereotyped object preferences appear to continue to increase in rigidity throughout early childhood. Halim et al. (2013) observed increased rigidity between the ages of 3 and 4 years, followed by constancy between the ages of 4 and 5 years. When investigating middle childhood, Hässler et al. (2022) encountered similar results. Toy preferences were identified as being particularly well correlated for transgender children and unrelated cisgender children between the ages of 7 and 9.5 years. This consistency also appears to continue into adolescence. Golombok et al. (2012) found continuity in participants' sex‐typed behaviour assessed at age 3 and age 13. As individuals approach adulthood, Kahn and Halpern (2019) observed another peak in gender‐typical preferences for participants aged 18–26. This was followed by a decrease in the final wave of the study. Overall, while the trajectory of object and activity preference does not appear to be a linear intensification process, the literature appears to evidence stability in the preferences of individuals.

Although these trends seem to be common among studies, individual differences have been observed. Campbell et al. (2000) and Shirley (2000) noticed that, at 3 months old, both sexes showed a preference for masculine toys and activities. Both authors hypothesized that this may be associated with the increase in moving features in these ‘male’ toys. As a whole group, participants showed sex‐congruent preferences by the age of 9 months but, when split into male and female results, these preferences were only significant in the male group. This preference for masculine toys intensified at 9 and 18 months for male infants. Evidence of more stereotypical preferences being demonstrated by boys was also collected by Behrendt (1989), Hässler et al. (2022) and McHale et al. (2004) in middle childhood.

### Clothing preferences

Clothing preference is used frequently throughout the research literature as a means of observing and measuring a child's gender expression. Specifically, 8 of the included studies used clothing preferences as part of their measures of gender identity. However, longitudinal data from the included studies indicates that a child's clothing preferences have a unique trajectory, distinct from other measures of gender identity (Bruun & Farr, 2020; Gülgöz et al., 2022; Halim et al., 2013; Hässler et al., 2022). Halim et al. (2013) observed this age to be a ‘peak’ in rigidity in children's clothing choices, followed by increased flexibility in the degree of gender conformity by age 4–5. In both qualitative parental accounts collected by Barron and Capous‐Desyllas (2017) and a quantitative study based on researcher observation conducted by Halim et al. (2013), children appeared to demonstrate gendered clothing preferences by the age of 3. This early ‘peak’ followed by increased flexibility was also observed by Bruun and Farr (2020). While children's gendered presentation was found to be associated with their parents' presentation at wave 1 of the study (mean age = 3 years), this did not persist into the second wave (mean age = 8.34 years). Bruun and Farr (2020) hypothesized that this increase in flexibility may be related to the age at which a child develops more autonomy over their clothing choice. It is also possible that increased flexibility coincides with developmental changes in the type of reasoning a child is able to use (Szkrybalo & Ruble, 1999).

### Sex segregation/peer preferences

Seven of the included studies measured sex segregation or peer preference alongside other measures of gender identity. Starting at 3 months of age, researchers such as Campbell et al. (2000) and Shirley (2000) used paired photographs of peers to assess whether infants showed same‐sex preferences. Campbell et al. (2000) found a small significant preference for same‐sex photos of peers at 3 months of age, but this was not replicated in later waves of the study, despite the increase in gendered cues demonstrated in photos of older children. The results collected by Shirley (2000) were also variable and did not evidence of sex segregation in infancy. However, when investigating the peer preferences of older children, Halim et al. (2013) found a curvilinear increase in sex segregation between the ages of 3 and 5. Within this study, 48% of 3‐year‐old children's friends, 70% of 4‐year‐old friends, and 74% of 5‐year‐old children's friends were found to be of the same gender. This sex segregation appears to remain stable over time, with Hässler et al. (2022) finding evidence of a significant positive correlation between children's peer preferences at age 7 and 9.5 years. Kornienko et al. (2016) found that adolescents tended to gradually develop similar levels of intergroup bias to that of their nominated friends.

### Comparison of trajectories in transgender and cisgender children and young people

When exploring the experiences of transgender children and young people, many researchers have found evidence to suggest that transgender children show very similar patterns of development to control groups of children of their expressed gender. Across a variety of measures, Rae et al. (2019) found that this similarity was present regardless of whether a transgender child had been able to socially transition. Although stronger cross‐sex identification was found to be predictive of later social transition (Rae et al., 2019) and persistence of gender dysphoria (Steensma et al., 2013), the scores that children obtained using these measures prior to transitioning did not differ significantly from those who had transitioned already or to a control group of their expressed gender (Rae et al., 2019). Hässler et al. (2022) also observed highly similar trajectories for transgender children, cisgender siblings, and unrelated cisgender children. The stability in gender identity displayed across these populations is reflected in the statistics around the persistence of gender identity in transgender individuals. As part of an ongoing longitudinal study, Potter et al. (2021) used a 4‐item gender survey to explore gender identity in 8‐ to 9‐year‐old children. Approximately 0.5% of children reported that they were transgender at time point 1, followed by 1% at time point 2. This was echoed in the data collected by Gülgöz et al. (2022), who found that children showed consistency in their use of a continuous measure of gender identity over the course of 1–2 years. Gülgöz et al. (2022) found that transgender children, their cisgender siblings, and unrelated cisgender children did not differ significantly in the way that they identified with their current gender. No group identified 100% with their current gender, indicating that binary measures may not accurately reflect the unique experience of individuals.

## DISCUSSION

Overall, the results of this systematic review are consistent with wider research suggesting that distinct developmental patterns can be observed depending on the measure of gender identity used (Egan & Perry, 2001). Prior to the time at which children can reliably identify their own gender and the gender of others, they seem to display gendered preferences (Campbell et al., 2000; Lauer et al., 2018). At this age, children do not appear to show any intergroup bias or same‐sex preference, yet they begin to develop a preference that adheres to the stereotypes associated with these groups (Campbell et al., 2000; Shirley, 2000). These preferences may be typical of the gender they have been assigned at birth, but this is not always the case (Bruun & Farr, 2020; Gülgöz et al., 2022). Across the included studies, the age of 3 appears to be a particularly salient time in which children are becoming increasingly consciously aware of gendered cues and norms (Bruun & Farr, 2020; Halim et al., 2013). At the age of 3, children appear to increase their rigidity in their gender‐stereotyped clothing preferences and start to show evidence of sex segregation (Halim et al., 2013). However, as they enter middle childhood, it seems that these children's preferences stabilize rather than continue to intensify (Halim et al., 2013) and sometimes reduce in rigidity (Ruble et al., 2007). Once children reach middle childhood, they appear to show stability across measures in various constructs of gender identity included in this review (Hässler et al., 2022; Kornienko et al., 2016).

The fact that children demonstrate gendered preferences prior to being able to label the gender they are or the gender of others, suggests that there is some essentialist quality to gender. Some modern theorists such as Fausto‐Sterling (2021), suggest that these preferences may be influenced by a combination of biological factors and a child's early experiences and interactions with adults. Fausto‐Sterling presents a developmental systems perspective in relation to gender identity development, based on the concept of ‘embodiment’. Embodiment is a biological theory that places emphasis on the interactions between an organism and its environment, particularly in the context of sensory‐motor activity. This model is used to explain how factors such as the physical touch and sensory interactions a child experiences between the ages of 0 and 18 months, may contribute to a child's emerging preferences and subjective sense of gender, prior to the time at which a child is able to internalize and consolidate their gender identity. Other research has also investigated more nuanced biological influences on gender. For example, neuroendocrinology (the study of hormones) has been used to assess the impact of hormonal differences on an individual's gender identity and presentation (Frisén et al., 2009; Gillies & McArthur, 2010; van Anders et al., 2011). However, whilst these more nuanced biological and environmental factors may impact and contribute to a child's gender identity development, research continues to evidence that some children's preferences do not conform to their gender assigned at birth (Bruun & Farr, 2020; Gülgöz et al., 2022).

With so many children demonstrating gender identities incongruent with biological sex, it appears that neither nature nor nurture can fully explain this essentialist quality. As children develop and learn about the societal expectations and stereotypes attached to gender, an individual's gendered experience seems to remain stable over time, even when this does not match cultural and societal expectations. Attempts to inhibit gender identity to fit within the prominent binary systems and structures can have a negative impact on psychological outcomes (Olson et al., 2015). This would suggest that both the ‘doing gender’ schools of thought and the ‘being gender’ schools of thought have a place when understanding the nuance of gender identity development in children and adolescents (Hyde et al., 2019). Perry et al. (2019) suggest that further research is needed to explore how biological factors interact with cognitive, social, and behavioural variables to impact gender identity.

Another key finding of this systematic review is that when children reach middle childhood their self‐identified gender tends to remain consistent over time (Hässler et al., 2022; Kornienko et al., 2016). Components of an individual's overall gender identity, such as their gendered expression or preferences, may reduce rigidity. However, overall summary judgements of gender identity tend to remain consistent from middle childhood to adolescence (Golombok et al., 2012; Kahn & Halpern, 2019). Research into cognitive development and the types of reasoning that children is able to access at different stages in their childhood may help to explain the variation in the trajectories of gendered expression (Szkrybalo & Ruble, 1999). For this reason, further research regarding the gendered cues and criteria that children use to make a summary judgement about their gender identity may also be useful. Understanding the relative salience of various attributes for these children may support researchers in recognizing the impact of child developmental factors on these trajectories (Perry et al., 2019). Barron and Capous‐Desyllas (2017) hypothesize that rigidity in gendered presentation may be linked to the importance that these children place on being accepted and ‘verified’ by society. Qualitative accounts of gender identity over time may also help researchers to understand this trajectory of rigidity.

### Limitations

A significant limitation of this review is that all studies were conducted in Westernized countries, which prevents consideration of any findings in relation to cross‐cultural differences in gender development. Similarly, a large number of studies were contextualized in North America which will have impacted the overall narrative of the data. Further research focusing on gender identity development in a broader range of cultures is needed. In addition to this, many studies within the adolescent and adult age ranges were not included in the current review due to exclusion criteria based on the idea of ‘intervention’. By this time in their lives, participants had often experienced various psychological or hormonal interventions. This means that this review is based on a large number of studies based on childhood and therefore provides limited evidence for the stability of gender identity in adulthood without the influence of these interventions. These exclusion criteria also had an impact on the range of research with transgender participants that it was possible to include, limiting the amount of direct comparison that it was possible to make between cisgender and transgender development for each of the types of measures in this review.

### Implications for practice

A clear implication for future practice in psychological research is the evidence that gender is a multidimensional construct, and the use of single dichotomous measures is not sufficient to fully understand an individual's gender identity. This has implications beyond the research literature and is particularly relevant when considering the operational definition of ‘gender’ used to create policies such as the UK Equalities Act. To develop systems and structures based on a single construct of gender identity, such as primary sexual characteristics, would disregard scientific evidence and risk further marginalizing individuals whose experiences fall outside of the binary (Hyde et al., 2019). Instead, future working definitions of gender should emphasize the multifaceted nature of gender, reflecting the complex interaction of social, cultural, psychological, and biological factors (Wood & Eagly, 2015).

In addition to this, this review provides evidence to suggest that an individual's self‐identified gender identity tends to remain stable over time, despite changes in rigidity in their gender expression (e.g., clothing, activity, or toy preferences). For this reason, future research should also include greater specificity regarding whether measures are based on gender expression, gender identity, or biological sex rather than simplifying the broader concept of gender to one of these components.

## AUTHOR CONTRIBUTIONS

**Ellena Fisher:** Writing – original draft; methodology; writing – review and editing; formal analysis; data curation; project administration; conceptualization. **Sarah Wright:** Writing – review and editing; supervision; conceptualization. **Cora Sargeant:** Writing – review and editing; supervision; conceptualization.

## CONFLICT OF INTEREST STATEMENT

The authors declare no conflict of interest related to this work.

## Data Availability

The data that support the findings of this study are available on request from the corresponding author. The data are not publicly available due to privacy or ethical restrictions.

## APPENDIX 1 DATA EXTRACTION TABLE

**Table:**

	Study	Participants	Time points	Methods/Measures	Data analysis	Main findings
1	Barron and Capous‐Desyllas (2017)	USA Purposeful sample 4 families from a transgender support group 5.5–8.5 years	Over the course of a year	Case study and ethnographic methods (participant observations, journals, interviews) 12 visits per household, and interviews for all family members	Cross case and comparative analysis Inductive coding Themes and subthemes	Gender non‐conforming generally began at age 2–3 All case study children enjoyed dressing up in preschool All children experienced a stage of fluidity, moving towards the constraints of the binary Children showed stereotypical gendered preferences and socially constructed ideas of gender Journeys were based on the choices of the children Parents felt a sense of blame from society The need to be accepted by society in daily interactions was very important to the children and may be associated with intensity of presentation
2	Behrendt (1989)	33 children at wave 1 aged between 4 and 9.5 yearRecruited from schools and preschools USA	Follow up after 4 years	Draw a person test Semi structured interview about gender	Coded responses Descriptive statistics	Girls were more consistent in their gender constancy Boys and girls responded differently Gender stereotypes hold meaning for children Girls referred more to internal personality characteristics, but boys referred more to external appearance characteristics Girls were less gender stereotyped
3	Bruun and Farr (2020)	106 families in the USA Adoptive parents: Lesbian, gay, and heterosexual Children 13–72 months old at time point 1	2 time points, 5 years apart	Standardised questionnaires and observational coding of gender presentation Parents and children unstructured play session with gender typical and gender‐neutral toys Interviews and family interaction tasks Inventory of parent and peer attachment (IPPA; Armsden & Greenberg,^1987^) Participants rated 1–5 in gender expression, gender conformity and gender non‐conformity	Bivariate correlation and one‐way MANOVA Power analyses	Children across family types were generally gender‐conforming in their presentation Children's conforming presentation in early childhood was not related to conforming behaviour in middle childhood. Children's gender presentation and friendship quality were not found to vary based on parent sexual orientation Parents’ presentation was not significantly associated with child presentation. Gender presentation follows a different developmental trajectory than gender‐typed attitudes, behaviours, and traits
4	Campbell et al. (2000)	60 infants, 3–18 months UK	3, 9, and 18 months	Sex‐congruent and sex‐incongruent paired stimuli from four domains (baby faces, child faces, toys and activities) Rouge mirror test Duration of gaze to each stimulus was measured	Photos taken coded by frame and converted into units of time to calculate duration of gaze Two‐way ANOVA	No evidence of self‐recognition by 18 months No consistent same‐sex peer preference across age ranges Sex‐congruent toy choice by 9 months of age Sex congruence significant in male but not female children Both sexes showed a preference for ‘masculine’ activities Activity preference remained stable over testing ages
5	DiDonato et al. (2012)	Participants recruited from a larger longitudinal study USA 115 preschool students	Twice weekly over the course of an academic year	Observational coding of naturally occurring gendered behaviour Average of 131 observations per child Gender typicality assessed through activity choices and peer play Adjustment measures: Child behavioural checklist (teacher report)	Bayesian Probability Score Hierarchical regression	Well‐adjusted children showed flexible gendered behaviour to suit different contexts Only when behaviour was more random that it was linked to adjustment Support for both consistency and flexibility hypotheses Encouraging children to be flexible in their gendered behaviour may increase adaptivity
6	Golombok et al. (2012)	UK, Bristol, Large ALSPAC study Sample based on PSAI scores at age 3.5, *N* = 377 6 groups of children: (1) extremely masculine boys, (2) extremely masculine girls, (3) extremely feminine girls (4) extremely feminine boys, (5) randomly selected boys, (6) randomly selected girls	Follow up at age 13 55% of the age 3 sample	Sex‐typed behaviours measured at 3.5 yeas using PSAI (Preschool Activities Inventory) – parent report Multidimensional Gender Identity and Sexual Questioning Scale administered at age 13 – child self‐report	MANOVAs of boys and girls separately	Findings showed continuity in sex‐typed behaviour from preschool to adolescence Girls classified as masculine at age 3, felt less gender typicality, less contentedness, and more self‐efficacy for male sex‐typed activities at age 13 Boys classified as feminine at age 3, felt less gender typicality and had lower self‐efficacy for male typed activities at age 13 in comparison to randomly selected boys (gender typicality, agentic traits, male typed activities) Girls classified as extremely masculine at age 3 varied on more subscales at age 13. Greater variation in sex‐typed behaviour in girls. Evidence more compatible with hormonal and social influence than a cognitive‐developmental theory of gender
7	Gülgöz et al., 2022	Participants from larger longitudinal study 4 groups (1) transgender, (2) gender non‐conforming, (3) cisgender siblings of trans or non‐conforming children and (4) unrelated cisgender children USA and Canada Study 2 = 196 children, 6–9 years	2 testing sessions (*M* = 1.95 years apart)	Gender spectrum task using a continuum from ‘feeling totally like a boy’ to ‘feeling totally like a girl’ – percentage Toy, clothing and peer preferences measure (child report) Similarity task – 10 questions (5‐point scale) Gender identity implicit association test Categorical gender identity – children asked to describe identity	Study 1: One way ANOVA examining effect of participant group on spectrum scores Study 2: Independent samples t tests	Transgender children, cis siblings and unrelated cis children did not differ from one another in the extent they identified with their current gender on the continuous measure Gender non‐conforming children showed greater variability across the scale None of the groups identified exclusively with their own gender – continuous measure needed Even cis and trans children did not score themselves as 100% their current gender Participant scores on the spectrum measure were moderately stable over time
8	Halim et al. (2013)	Preschool children (*N* = 229) and their mothers 154 families participate in all 3 waves of the studies Mexican, Dominican, or US born African American mothers.	3 waves Ages 3,4, and 5	Observations of gender‐typed appearance coded at each stage (masculine or feminine) Gender typed dress‐up play – reported by mothers on a 4‐point scale Gender‐typed play – reported by mothers on 4‐point scale for feminine and masculine items Sex segregation – mothers asked to list up to 9 peers their child spends time with and their sex (siblings excluded)	Multilevel models of behaviour Gender and ethnicity as factors and time as a covariate Structural equation model to assess stability across time	Nearly all behaviours moved towards greater rigidity over time (dress‐up, gender‐typed play, sex segregation increased between ages of 3 and 4). Sex segregation increased from ages 4 to 5 Dress up and gender typed play remained stable, but cross gender typed play decreased Observed gender‐typed appearance remained stable from age 3 to 4 Gender typed appearance decreased in rigidity from age 4 to 5 Ethnicity generally did not affect scoring Gender dimensions showed distinct patterns Moderate stability in individual gendered behaviours from year to year
9	Hässler et al. (2022)	Large ongoing longitudinal study (*n* = 433). USA Included first testing session and the most recent testing session. Mean ages 7 and 9	Two time points, an average of 2.6 years apart.	Gender Identity Measure: ‘Do you feel like you are a boy, girl, or something else?’ and ‘When you grow up, do you think you will feel like a boy, a girl, or something else?’, Gender Similarity: five questions about how similar they are to boys and five questions about how similar they are to girls, answers on a 5‐point scale Toy Preference Task Peer Preference Task Clothing Preference Task Composite Score	Preacher's procedure Linear mixed model for repeated measures	Consistency across the 2 and a half year time span across measures for cis and transgender children Effect size different depending on measure Toy preferences particularly well‐correlated over time Clothing preferences less consistent over time in all groups Trajectories were highly similar across the three groups of children Overall trend toward greater rigidity in gender development among boys than girls
10	Kahn and Halpern (2019)	20,745 adolescents in 7th–12th Grade as part of national study USA	Tested in 1995, 1996, 2001, 2008	Biological Sex Sexual Orientation (self‐report in wave 4 interview) Gender Typed Behaviour – (AGB) self‐reported at each wave	Multilevel mixed regression models to assess longitudinal variation	Longitudinal analysis showed that gender‐typed behaviour increased from adolescence to early adulthood, but that this trajectory was not linear. Generally, participants became more gender conforming in waves 1–3 and then less conforming again in later waves Gender intensification peaked at emerging adulthood (18–25) In all groups, scores spanned the measure suggesting that gender non‐conforming is common in all groups, not just those with minority sexual orientation Results suggest that gender typed behaviours do not predict sexual orientation
11	Kornienko et al. (2016)	Part of a wider longitudinal study on identity development *N*‐ 670 aged 12–13 USA	Wave 2 and 3 of larger study Age 12 and 13	Self‐Report Survey with questions about identity, psychological and educational outcomes Friendship nominations Gender Identity – self‐identification as well as 4 continuous measures (1) gender typicality, (2) felt pressure from peers for gender conformity, (3) intergroup bias (4) gender contentedness Friendship networks – asked to nominate 10 friends so that researchers could form friendship networks matrices	Stochastic Actor‐Based Modelling	Peer socialisation affected between‐gender dimensions of GI: intergroup bias and felt pressure for gender conformity Same sex preferences demonstrated in both year groups Over the course of the school year, both year groups showed changes in their levels of intergroup bias and felt pressure to conform to become similar to the levels of their friends Between‐gender group dimensions of GI might be more susceptible to peer influence than the within‐gender dimensions of GI
12	Lauer et al. (2018)	52 children (25 girls) USA	Time A: 6–13 months Time B: 4 years old	Object preference task at 6–13 months Parent Questionnaire about gendered preferences at age 4 Gendered play score calculated for each child	Steiger's (1980) *z* tests	Evidence of developmental stability in gendered preferences across early childhood Object preferences in 1st year of life are precursors for future play preferences
13	Martin, 2010	Cohorts of 3‐ and 4‐year‐old children as they joined Nursery and moved into Reception classes UK	Over 2 years	Participant observation Ongoing field notes based on discussions with staff and parents Child interviews Play/conversations/preferences	Ethnographic and discourse analysis Grounded Theory	Children learned norms of gender behaviour from each other Gender stereotypes were policed and regulated by the children Heteronormativity was sustained through classroom activities Children were influenced by ‘more established’ and older children Boys learned to disassociate themselves from femininity Children were keen to demonstrate their ‘correct’ gender positioning Adults provide important role models
14	McHale et al. (2001)	Longitudinal study about gender role socialization in middle childhood 198 families participated in current study USA		Interviews with parents/1st and 2nd born siblings including attitude to gender role measures for children and parents and parent sex‐typed leisure activities measure 7 evening telephone interviews to ask about child's activities	Mixed model ANOVA	Pattern of second born sibling gender role orientations consistent with social learning from 1st born sibling Firstborns followed a pattern that suggested they changed to distinguish themselves from siblings (de‐identification) More evidence for sibling influence than parent influence Cognitive developmental changes mean that children become more flexible in their ideas about gender Girls demonstrated more flexible attitudes than boys Second born children less stereotypical than first born children
15	McHale et al. (2004)	Longitudinal study about gender role socialization in middle childhood 200 families participated in current study USA	Time 1: 10.81 years Time 2: 12.82 years of age	Interviews with families – each member separately asked about personal qualities and family relationships Children attitude towards women scale Sex typed personality qualities measure Interest in languages and maths Self‐esteem scale 7 evening telephone interviews to ask about child's activities	Correlational analysis	Consistent sex differences in the ways in which and the people with whom children spent their time Sex typing was more apparent in boys’ time with peers Girls less stereotyped when with female peers
16	Potter et al. (2021)	Ongoing, longitudinal, US cohort Study *N* = 4, 95 9–10 years USA	1 year follow up visit	4‐item gender survey measuring felt‐gender, gender noncontentedness, and gender nonconformity Youth KSADS‐5 suicide module (Kaufman et al., 1997) Child Behavior Checklist (CBCL) (Achenbach, 2009) Puberty Development Scale (PDS; Petersen et al., 1988)	Logistic regression Linear mixed models	Prevalence of transgender children at 9 years was 0.5% and then 1% at 10 years Females showed greater gender diversity than males. 39.5% of 9/10‐year‐olds respond ‘I don't understand’ when asked if they are transgender
17	Rae et al. (2019)	Cohort of gender‐nonconforming children (*n* = 85) compared with groups of transitioned transgender children (*n* = 84) and gender‐conforming controls (*n* = 85). Recruited from community groups USA	2 year follow up	Child self‐report Five gender‐development measures: peer preference (from Olson et al., 2015), toy and clothing preference (from Fast & Olson, 2018), gender similarity (Martin et al., 2017), and gender identity (Fast & Olson, 2018). Composite score calculated	Logistic regression model	Stronger cross‐sex identification and preferences at initial testing predicted social transitions Children who later transitioned scored similarly to those who had already transitioned and to the control group of their expressed gender Social transition does not appear to impact preferences and identification
18	Shirley (2000)	60 infants recruited by health visitors and media advertising UK	3, 9, and 18 months	Duration of attention paid to male or female stereotyped stimuli presented in pairs Rouge mirror test to investigate self‐identification and time looking at own picture/picture of same age/sex child Gender labelling assessment at 18 months	T‐tests ANOVA Cross domain stability measure	No clear pattern of sex typed behaviour at 9 months Infants could self‐identify at 18 months but were not accurate in the gender labelling assessment task Same sex preferences for peers and play activities shown in male infants but not in group as a whole (with female infants included) Significant sex‐congruent toy preference by 18 months
19	Steensma et al. (2013)	127 adolescents (79 boys, 48 girls), who were referred for GD in childhood (<12 years of age) The Netherlands	4‐year period	WISC Gender Identity Interview for Children (GIIC) Gender Identity Questionnaire (GIQ) – Parent report Child Behavior Checklist Peer Relations Scale Gender Identity Interview for Adolescents and Adults (GIAA) Utrecht Gender Dysphoria Scale (UGDS) Body Image Scale (BIS) Sexual Orientation Questions	Bivariate logistic regressions	Factors associated with persistence vary among natal boys and girls Persisters reported higher intensities of GD, more body dissatisfaction, and higher reports of a same‐sex sexual orientation compared to desisters Link between the intensity of GD in childhood and persistence of GD Higher probability of persistence among natal girls Psychological functioning and the quality of peer relations did not predict the persistence of childhood GD
20	Szkrybalo and Ruble (1999)	Preschool, kindergarten, and lst‐grade children (*N* = 195) Part of a large longitudinal study USA	3‐year period	Sex‐category constancy measure (derived from Slaby and Frey, 1975, Wehren and de Lisi, 1983 and Frey and Ruble, 1992)	Child responses coded into explanation categories ANOVA	Constancy performance appears to improve or decline over time depending on the kind of reasoning that children use at different ages to explain sex‐category membership Children's SCC judgments showed a clear increase from ages 6 to 8 Children are more likely to cite stereotypes as reasons for a person's sex identity to change than as reasons for it to remain constant (e.g., boys can cook too) Sequence of self‐constancy before other constancy
21	Warin (2000)	10 children during the transition to School UK	6 months prior to school attendance to end of second year of schooling	The pictorial scale of perceived competence and social acceptance Harter and Pike (1984); Semi structured interview about toy, activity and peer preferences Slaby and Frey (1975) gender understanding questions Measure of the child's desire for gender consistency (clothing task)	Mixed methods	Range of understanding across the 10 children at first testing for self‐consistency measure Association between a lack of gender constancy and a preparedness to engage in non‐conforming behaviour 6 gender‐constant children appeared to be much more gender‐stereotypical in their preferences
